# A mixed methods study of siblings’ roles in maternal feeding practices in early childhood: an application of the learning from experience process

**DOI:** 10.1186/s12966-022-01302-3

**Published:** 2022-06-07

**Authors:** Cara F. Ruggiero, Amy M. Moore, Michele E. Marini, Stephen R. Kodish, Susan M. McHale, Jennifer S. Savage

**Affiliations:** 1 Center for Childhood Obesity Research, The Pennsylvania State University, University Park, PA USA; 2 Department of Nutritional Sciences, The Pennsylvania State University, University Park, PA USA; 3 Department of Biobehavioral Health, The Pennsylvania State University, University Park, PA USA; 4 Department of Human Development and Family Studies, The Pennsylvania State University, University Park, PA USA

**Keywords:** Responsive feeding, Siblings, Family systems, Mixed methods, Infancy, Toddlerhood

## Abstract

**Background:**

Firstborn children have higher rates of obesity compared to secondborns, perhaps due, in part, to differential feeding practices. Despite the centrality of siblings in family life and potential for influence, almost nothing is known about the role of siblings in parent feeding practices in early childhood.

**Methods:**

Participants (*n* = 117) were mothers of consecutively born siblings. Firstborns participated in an RCT that compared a responsive parenting intervention designed for primary prevention of obesity against a safety control. Secondborns participated in an observational cohort. Multilevel models tested whether and how firstborn characteristics (temperament, appetite, rapid weight gain) at 16 weeks and 1 year were associated maternal feeding practices of secondborns in infancy at 16 weeks, 28 weeks, and 1 year (food to soothe) and at ages 1, 2, and 3 years (structure-and control-based feeding practices). A purposive subsample (*n* = 30) of mothers also participated in semi-structured interviews to further illuminate potential sibling influences on maternal feeding practices during infancy and toddlerhood.

**Results:**

Firstborn characteristics did not predict secondborn feeding in infancy (all *ps* > 0.05). Firstborn negative affect, however, predicted mothers’ less consistent mealtime routines (*b* (SE) = − 0.27 (0.09); *p* = 0.005) and more pressure (*b* (SE) = 0.38 (0.12); *p* = 0.001). Firstborn appetite predicted mothers’ less frequent use of food to soothe (*b* (SE) = − 0.16 (0.07); *p* = 0.02) when secondborns were toddlers. Firstborn surgency, regulation, and rapid weight gain, however, did not predict secondborn feeding practices during toddlerhood (all *ps* > 0.05). Interviews with mothers revealed three ways that maternal experiences with firstborns informed feeding practices of secondborns: 1) Use of feeding practices with secondborn that worked for the firstborn; 2) Confidence came from firstborn feeding experiences making secondborn feeding less anxiety-provoking; and 3) Additional experiences with firstborn and other factors that contributed to secondborn feeding practices.

**Conclusions:**

Some firstborn characteristics and maternal experiences with firstborns as well as maternal psychosocial factors may have implications for mothers’ feeding practices with secondborns. Together, these mixed methods findings may inform future research and family-based interventions focused on maternal feeding of siblings in early childhood.

## Background

Firstborn children have a higher prevalence of obesity than their younger siblings [1–5], despite secondborn siblings experiencing a prenatal environment characterized by higher maternal pre-pregnancy Body Mass Index (BMI), risk for gestational diabetes, and subsequent higher birthweight [6]. Postnatal factors such as how and what infants are fed, the unique individual characteristics of each sibling, and shifts in family dynamics with the birth of the secondborn may partially explain this finding [7]. National U.S. data indicate that approximately 80% of children < 18 years old have at least one sibling [8]. Nonetheless, most obesity research focuses on one child per family [9], and little is known about sibling differences in feeding [7, 10]. The parenting literature suggests that siblings may influence one another indirectly by their impacts on family structure (size, gender constellation) and dynamics (e.g., family roles, allocation of resources) [11].

A sibling-related family dynamic involves parents’ opportunities to learn from their experiences with firstborns [11]. Learned experience refers to the ways in which earlier-born children, by virtue of their behaviors and characteristics, may influence their parents’ knowledge and expectations for laterborn siblings-- in both positive and negative ways. For example, parents who experienced a child’s transition to adolescence as “easy” were less likely to expect transition difficulties in the child’s younger sibling [12]. Parents also exhibited more effective parenting, including higher levels of warmth and parental knowledge and lower conflict, with secondborn compared with firstborn adolescents. Despite the centrality of siblings in family life and potential for influence on family health and well-being, almost nothing is known about the role of siblings in parent feeding practices in early childhood.

One way to examine parent feeding practices in early life is by studying responsive feeding. Responsive feeding captures the structure, routine, and emotional context of feeding interactions and includes three components: 1) perception of the child’s cues (e.g., hunger; satiation), 2) accurate cue interpretation, and 3) appropriate cue responses [13, 14]. Structure-based practices such as rules and routines around mealtimes are considered to be responsive [15] and promote healthy child weight [16, 17]. On the other hand, non-responsive, control-based feeding practices such as using food to soothe non-hunger related distress increases risks for overeating, weight gain, and obesity later in childhood [18–21]. A body of work documents that child characteristics such as temperament are linked to parents’ feeding practices. For example, parents use more nonresponsive, control-based parent feeding practices such as food to soothe when they perceive their infants to be reactive or fussy (i.e., negative temperament) [22, 23]. Parental concern about child overweight is also a consistent predictor of feeding practices [24]. Further, child appetitive traits (e.g., food responsiveness, satiety responsiveness) have been linked to parent feeding practices [25–28] and subsequent child weight [29].

A next step in this research is to determine whether such children’s characteristics have implications for parents’ feeding practices later on with younger siblings. For example, if mothers learn from experiences to use food to soothe with fussy firstborns, they may use the same obesogenic feeding practices with secondborns regardless of the secondborns’ characteristics, because these practices were effective in the past. In addition to child characteristics, maternal psychosocial factors such as stress and parenting satisfaction also have been shown to influence feeding practices [30–34]. For example, lower maternal self-esteem has been associated with greater feeding pressure [31], and maternal stress, anxiety, and depression have been linked to nonresponsive feeding styles [30]. These psychosocial factors may provide the larger context within which mothers apply what they have learned from experiences with earlier-born children to laterborn siblings.

Building on prior literature, in this analysis, we used a mixed methods approach to address two aims.

- Aim 1. To test whether, consistent with a learning from experience process, firstborn characteristics predict mothers’ feeding practices with secondborns such that, (Hypothesis 1.i) mothers of firstborns with “difficult” temperaments in infancy use more control-based feeding and less structure-based feeding with secondborns; (Hypothesis 1.ii) mothers of firstborns with larger appetites use more control-based feeding and less structure-based feeding with secondborns; and (Hypothesis 1.iii) mothers of firstborns with faster weight gain use less control-based feeding and more structure-based feeding with secondborns.
- Aim 2. Elaborating on the quantitative results from Aim 1, to explore mothers’ perspectives on factors that influenced their feeding of secondborns, particularly their experiences with firstborns.

## Methods

A sequential explanatory, mixed methods design was used to explore whether and how mothers learned from their feeding experience with firstborns [35]. Quantitative analyses assessed the association between firstborn characteristics and feeding practices of secondborns. Data from retrospective semi-structured interviews conducted with a subset of mothers were then used to contextualize the quantitative findings. At the time of the interviews, firstborns and secondborns in this subset were on average 8.3 and 5.9 years of age, respectively. The interview questions (Table 1) allowed for an in-depth exploration of key themes surrounding *what* and *how* mothers learn to feed their laterborn children. This study was approved by the Human Subjects Protection Office of the Penn State College of Medicine.

**Table 1:** Selected Semi-Structured Interview Guide Questions

**Introduction Questions**	
1. How would you describe your firstborn when he/she was a baby? • *Probe on temperament, appetite, growth*	
2. How would you describe your secondborn when he/she was a baby? • *Probe on temperament, appetite, growth*	
**Learned Experience**	
3. Could you first explain how you learned how to feed your children during infancy? • *Probe on how they learned to feed firstborn (*e.g.*, family members)* • *Probe on how they learned how to feed secondborn (*e.g.*, experience from firstborn)*	
4. What specifically did you learn from feeding your firstborn during infancy that may have influenced how you fed your secondborn during infancy? • *Probe on spacing making it easier or more difficult* • *Probe on firstborn characteristics (*e.g.*, temperament, appetite, growth)*	
5. Can you tell me about using food to calm or soothe with your children in infancy? • *Probe on food to soothe with firstborn and secondborn* • *Probe on whether what they learned worked well with firstborn and used with secondborn* • *Probe for specific narratives/stories explaining why*	
6. Can you tell me about using food to reward behavior or as a bribe with your children in toddlerhood? • *Probe on use with secondborn—did you learn about it being effective because of your firstborn?*	
7. Could you first explain how you learned how to feed your children during toddlerhood? • *Probe on how they learned to feed firstborn (*e.g.*, family members)* • *Probe on how they learned how to feed secondborn (*e.g.*, experience from firstborn)*	
8. What specifically did you learn from feeding your firstborn as a toddler that may have influenced how you fed your secondborn during toddlerhood (1 to 3 years old)? • *Probe on spacing making it easier or more difficult* • *Probe on firstborn characteristics (*e.g.*, temperament, appetite, growth)*	

### Participants

The *Intervention Nurses Start Infants Growing on Healthy Trajectories* (INSIGHT) study is a randomized clinical trial comparing the effects of an intervention designed to promote responsive parenting toward preventing childhood obesity in firstborn infants versus a safety control intervention among 279 primiparous mother-infant dyads [16, 17]. Maternal eligibility criteria included English speaking, at least 20 years old, and residing within 50 miles of the university’s medical center. Firstborn infants had to be full-term, healthy, and singleton with a birthweight of ≥2500 g. Details on study design have been previously published [36].

Mothers participating in INSIGHT were invited to participate in an observation-only study involving secondborn siblings, SIBSIGHT, following the birth of their second child (*n* = 117). Inclusion criteria for the secondborn included singleton infants ≥36 weeks of gestation, no medical conditions that would impact feeding, birth weight ≥ 2250 g, and families planning to live in the study region for 1 year following the birth of their secondborn. The quantitative analyses included 117 mothers who participated in INSIGHT and SIBSIGHT. In addition, a purposive sample utilizing maximum variation [37] of INSIGHT mothers with a secondborn child who participated in SIBSIGHT (*n* = 30) was recruited to participate in semi-structured interviews aimed at exploring the implications of sibling-related processes as well as other factors identified in prior work (temperament, appetite, rapid weight gain) on maternal feeding practices during infancy and toddlerhood. Our sampling framework included prioritizing contacting mothers who had sibling dyads who were discordant on a number of characteristics such as sex, temperament, appetite, and rapid weight gain as well as variability in other characteristics such as birth spacing and study group.

### Measures

#### INSIGHT trial in firstborns

*Demographic information* was collected at enrollment. Race/ethnicity was collected using categories consistent with National Institutes of Health enrollment tables. Maternal age, pre-pregnancy weight, infant sex, and gestational age were extracted from medical records.

Firstborn infant *temperament* was assessed using the Infant Behavior Questionnaire-Revised Very Short Form (IBQ-R VSF) [38] and the Infant Behavior Questionnaire-Revised (IBQ-R) at firstborn age 16 weeks and 1 year, respectively. Mothers rated the frequency infant behaviors using a response scale that ranged from 1 = Never to 7 = Always. Three subscales were used in the analyses: surgency (i.e., extraversion, α = 0.76 at both timepoints), negative affect (i.e., fussiness, α = 0.81, 0.83), and regulation (i.e., self-regulation, α = 0.72, 0.74).

Firstborn *appetitive traits* were assessed using the Baby Eating Behavior Questionnaire (BEBQ) [39] at firstborn age 44 weeks using one item during the period of milk feeding “My baby had a big appetite,” with response options ranging from 1 = Never to 5 = Always.

Firstborn *rapid weight gain* was assessed using anthropometrics collected by trained research nurses at birth and at firstborn age 28 weeks. Infant weights were obtained using a digital scale (Seca 354 and/or Seca 874). Recumbent lengths were obtained using an infant measuring board (i.e., Shorr Board). Infant weights and lengths were used to derive weight-for-age and length for age *z*-scores using World Health Organization reference standards [40]. Rapid weight gain from birth to 28 weeks in firstborns was measured in terms of conditional weight gain scores [41], calculated as standardized residuals from the linear regression of weight for age at 28 weeks on weight for age at birth, with length for age at birth and 28 weeks and infant age at the 28-week assessment entered as covariates. A conditional weight gain score of zero represents the population mean, with positive scores indicating faster-than-average weight gain, and negative scores indicating slower-than-average weight gain.

#### SIBSIGHT observational cohort in secondborns

Secondborn *food to soothe* in infancy was assessed using a modified version of the Baby’s Basic Needs questionnaire (BBN) [42] at secondborn age 16, 28, and 52 weeks. Mothers rated the frequency of using food to soothe on a scale from 1 = Never to 5 = Always. Subscales used in this analysis include contextual-based (α range = 0.77–0.83) and emotion-based (α range = 0.90–0.94) food to soothe.

Secondborn* Structure-and control-based feeding practices* (e.g., limiting exposure to unhealthy foods/routines and pressure/restriction respectively) with secondborns at ages 1, 2, and 3 years were assessed using the Structure and Control in Parent Feeding (SCPF) questionnaire [43] completed by mothers. Response options ranged from 1 = Never to 5 = Always and were averaged to create 4 subscales: limiting exposure to unhealthy foods (α range = 0.73–0.79), consistent mealtime routines (α range = 0.79–0.80), pressure (α range = 0.75–0.83), and restriction (α range = 0.63–0.73).

Secondborn* instrumental feeding* was assessed via maternal ratings on the Feeding to Manage Child Behavior (FMCB) questionnaire at secondborn ages 1, 2, and 3 years [44]. Response options ranged from 1 = Never to 5 = Always and were averaged to create two subscales: use of food to soothe (α range = 0.74–0.89) and food as reward (α range = 0.75–0.80).

A *semi-structured interview guide* was developed to retrospectively capture mothers’ experiences on how they applied what they had learned in feeding their firstborn to feeding their secondborn. The interview guide was developed by research team members with expertise in parent feeding practices and qualitative research (Table 1). Research on the learned experience process, as well as other factors linked to maternal feeding practices, informed guide development. Mothers were asked questions pertaining to how they learned to feed their children in infancy and toddlerhood, and what specifically they learned by feeding their firstborn during infancy that may have influenced how they fed their secondborn. These qualitative interviews built on prior research that has used a retrospective report approach to study feeding practices [45, 46] and qualitative literature in other fields showing that real time and 1-year retrospective accounts are similar in content, tone, and quality, suggestive of considerable rumination [47]. Interviews were digitally recorded and conducted virtually over Zoom [48, 49] in English by two trained researchers (CR, AM). Both researchers met weekly to discuss detailed field notes [50], to refine interview procedures, and determine saturation (i.e., a repetition of key information, at which point no additional data collection was thought to yield new insights relevant to the research question) [51], which was reached at 30 participants [52].

### Analyses

To test the learning from experience hypotheses, the quantitative analyses used a multilevel modeling approach (SAS PROC MIXED, SAS version 9.4, SAS Institute, Carey, NC) to test whether firstborn characteristics predicted secondborn feeding. Secondborn age in terms of measurement time point (categorical) was treated as the metric of time. In the first set of models, food to soothe scores at secondborn ages 16, 28, and 52 weeks were entered at Level 1 (within-person over time); in the second set of models, measures of strucure and control-based and instrumental feeding at secondborn ages 1, 2 and 3 years were entered at Level 1. At Level 2 (between-family level), firstborn characteristics (temperament at 16 weeks and 1 year, for infancy and toddlerhood models respectively, appetite at age 44 weeks and conditional weight gain from birth to 28 weeks) were entered as predictors of secondborn feeding practices across infancy and toddlerhood. All models were adjusted a priori for study group and secondborn characteristics (temperament, appetite, conditional weight gain, in separate models). Sibling sex constellation (same vs. different biological sex) was entered at Level 2 in each model and retained if significant. Birth spacing, secondborn age and study group also were included as covariates at Level 2 and tested as potential moderators of the links between firstborn characteristics and secondborn feeding. Mixed models using maximum likelihood estimation are robust in correcting for missing data [53, 54].

Analyses of the semi-structured interview data followed a multi-step process. First, audio-recorded interviews (*n* = 30) were transcribed verbatim and checked for accuracy by trained members of the research team. Second, transcripts were uploaded to Dedoose software [55] for coding using a content analysis approach [56–58]. This approach allowed for using a priori categories reflective of the learned experience process (deductive), while allowing for some emergent themes during the coding process (inductive). Third, two members of the research team (CR, AM) independently coded two of the same interview transcripts using a line-by-line approach that allowed codes to be generated from the data that were not initially included in the codebook. Fourth, inter-coder reliability (ICR) testing was conducted to ensure agreement between the two coders [59]. Pooled Cohen’s kappa of 0.87 indicated ‘almost perfect’ reliability [57, 60]. Fifth, the first 20% of transcripts (6/30) [59] were coded by both coders, and ICR was re-tested with ‘almost perfect’ reliability (0.93) [60]. The codebook was refined iteratively throughout steps one through five after discussions between the two researchers. Sixth, the remaining 80% (24/30) transcripts were coded by the first author. During the analytic process, the research team held weekly meetings for discussion related to coding challenges and questions to ensure continual alignment. Lastly, major concepts were identified, defined, and refined into main themes.

## Results

### Preliminary analyses

Mothers were predominantly white, non-Hispanic, married and had incomes >$50,000 (Table 2). Secondborns were born 30.2 ± 10.2 months after firstborns. There were 29.5 ± 9.9 months between the earliest measurements in firstborn characteristics and earliest secondborn feeding measures. No differences in baseline characteristics by study group were found. Mothers had higher pre-pregnancy BMIs with their secondborns. Firstborn infants had higher gestational ages than secondborn infants. Participant demographics did not vary between the mothers who did and did not complete the semi-structured interviews.

**Table 2:** Demographic characteristics of mothers and consecutively born siblings at the time of delivery (*n* = 117)

	Firstborn	Secondborn
**Maternal Characteristics**		
Pre-pregnancy BMI, kg/m2^a^	25.1 (5.1)	25.9 (5.5)^a^
Non-Hispanic white, *n* (%)	109 (93.2)	109 (93.2)
Married, *n* (%)	107 (91.5)	111 (94.9)
Household income, n (%)	–	–
< $50,000	17 (14.5)	8 (6.8)
≥ $50,000	97 (82.9)	101 (86.3)
Don’t know/refuse to answer/missing	3 (2.6)	8 (6.8)
**Infant Characteristics**		
Birthweight, kg, mean (*SD*)	3.4 (0.4)	3.5 (0.4)
Sex, female, *n* (%)	62 (53.0)	67 (57.3)
Gestational age at delivery (weeks)	39.6 (1.3)	39.4 (1.0)^a^
Responsive Parenting (RP) Intervention Group, *n* (%)^b^	57 (48.7)	57 (48.7)
Birth spacing, mean (*SD*), months	–	30.2 (10.2)

^a^Significant sibling differences

^b^There were no significant differences in demographic characteristics by study group

### Links between firstborn characteristics and secondborn feeding: quantitative results

Firstborn temperament, appetite, and rapid weight gain did not predict food to soothe in secondborns during infancy (all *ps* > 0.05; Table 3). In other words, we found no evidence of a learned experience process in secondborn feeding in infancy. In contrast, as shown in Tables 4 and 5, some firstborn characteristics predicted secondborn feeding during toddlerhood, evidence consistent with a learned experience process. Specifically, firstborn higher negative affect predicted mothers’ less consistent mealtime routines (*b* (SE) = − 0.27 (0.09); *p* = 0.005) and more pressure to eat (*b* (SE) = 0.38 (0.12); *p* = 0.001) in secondborn feeding over a year later. Also, firstborns with larger appetites predicted less maternal food to soothe with secondborns (*b* (SE) = − 0.16 (0.07); *p* = 0.02). Similar to findings in infancy, firstborn surgency, regulation, and rapid weight gain did not predict maternal feeding of secondborns during toddlerhood (all *ps* > 0.05).

**Table 3:** Results from multilevel models examining firstborn characteristics and maternal feeding of secondborns ^a^

	Contextual-based Food to Soothe	Emotion-based Food to Soothe
	Estimate (SE)	Estimate (SE)
**Model 1 Negative Affect (*n*** **= 105)**		
Intercept	2.35 (0.36)***	1.65 (0.38)***
Measurement time point (Secondborn age in weeks)	−0.10 (0.08)	− 0.005 (0.09)
FB Characteristic	−0.11 (0.07)	− 0.08 (0.08)
**Model 2 Surgency (*n*** **= 105)**		
Intercept	2.78 (0.40)***	1.80 (0.41)***
Measurement time point (Secondborn age in weeks)	−0.10 (0.08)	− 0.01 (0.09)
Firstborn Characteristic	0.03 (0.08)	−0.02 (0.08)
**Model 3 Regulation (*n*** **= 105)**		
Intercept	2.87 (0.62)***	2.53 (0.63)***
Measurement time point (Secondborn age in weeks)	−0.10 (0.08)	− 0.01 (0.09)
FB Characteristic	−0.07 (0.10)	− 0.16 (0.10)
**Model 4 Appetite (*n*** **= 91)**		
Intercept	2.13 (0.48)***	1.40 (0.50)**
Measurement time point (Secondborn age in weeks)	−0.11 (0.08)	− 0.02 (0.11)
Firstborn Characteristic	−0.09 (0.07)	− 0.08 (0.08)
**Model 5 Rapid Weight Gain (*n*** **= 116)**		
Intercept	2.48 (0.23)***	1.93 (0.23)***
Measurement time point (Secondborn age in weeks)	−0.11 (0.08)	− 0.02 (0.09)
Firstborn Characteristic	−0.04 (0.07)	− 0.09 (0.07)

† < 0.10,* < 0.05,** < 0.01,*** < 0.001

^a^Models adjusted for birth spacing, study group and FB characteristics

**Table 4:** Results from multilevel models examining firstborn characteristics and structure-and control-based feeding of secondborns^a^

	Limiting Exposure to Unhealthy Foods	Consistent Mealtime Routines	Pressure	Restriction
	Estimate (SE)	Estimate (SE)	Estimate (SE)	Estimate (SE)
**Model 1 Negative Affect (*n*** **= 100)**				
Intercept	3.64 (0.39)***	4.16 (0.36)***	0.88 (0.44)*	1.40 (0.60)*
Measurement time point (Secondorn age in years)	0.31 (0.04)***	0.12 (0.05)*	−0.28 (0.06)***	−0.32 (0.07)***
Firstborn Characteristic	−0.12 (0.10)	−0.27 (0.09)**	0.38 (0.12)**	0.01 (0.16)
**Model 2 Surgency (*n*** **= 100)**				
Intercept	3.75 (0.49)***	3.33 (0.47)***	2.94 (0.57)***	2.02 (0.78)*
Measurement time point (Secondborn age in years)	0.31 (0.04)***	0.12 (0.05)*	−0.28 (0.06)***	−0.32 (0.07)***
Firstborn Characteristic	−0.04 (0.12)	0.04 (0.12)	0.10 (0.14)	0.09 (0.19)
**Model 3 Regulation (*n*** **= 100)**				
Intercept	3.92 (0.50)***	3.18 (0.47)***	3.10 (0.58)***	2.96 (0.78)***
Measurement time point (Secondborn age in years)	0.31 (0.04)***	0.12 (0.05)*	−0.28 (0.06)***	−0.32 (0.07)***
Firstborn Characteristic	−0.006 (0.11)	0.15 (0.10)	−0.07 (0.13)	−0.11 (0.17)
**Model 4 Appetite (*n*** **= 90)**				
Intercept	3.48 (0.35)***	3.41 (0.32)***	2.22 (0.38)***	1.88 (0.51)***
Measurement time point (Secondborn age in years)	0.34 (0.04)***	0.15 (0.04)**	−0.31 (0.07)***	−0.36 (0.08)***
Firstborn Characteristic	0.04 (0.06)	0.06 (0.05)	−0.04 (0.06)	0.08 (0.08)
**Model 5 Rapid Weight Gain (*n*** **= 116)**				
Intercept	3.81 (0.15)***	4.03 (0.14)***	2.03 (0.18)***	2.63 (0.23)***
Measurement time point (Secondborn age in years)	0.29 (0.04)***	0.11 (0.05)*	−0.30 (0.06) ***	−0.35 (0.07)***
Firstborn Characteristic	0.03 (0.05)	−0.003 (0.05)	0.01 (0.06)	0.10 (0.07)

† < 0.10,* < 0.05,** < 0.01,*** < 0.001

^a^Models adjusted for birth spacing, study group and FB characteristics

**Table 5:** Results from multilevel models examining firstborn characteristics and instrumental feeding of secondborns ^a^

	Food to Soothe	Food as Reward
	**Estimate (SE)**	**Estimate (SE)**
**Model 1 Negative Affect (*n* = 100)**		
Intercept	1.23 (0.52)*	1.43 (0.55)*
Measurement time point (Secondborn age in years)	−0.18 (0.06)**	−0.86 (0.08)***
Firstborn Characteristic	0.19 (0.14)	0.16 (0.15)
**Model 2 Surgency (*n* = 100)**		
Intercept	2.06 (0.66)**	1.87 (0.70)**
Measurement time point (Secondborn age in years)	−0.18 (0.06)**	−0.86 (0.08)***
Firstborn Characteristic	0.23 (0.17)	0.16 (0.18)
**Model 3 Regulation (*n* = 100)**		
Intercept	2.35 (0.68)***	2.90 (0.71)***
Measurement time point (Secondborn age in years)	−0.18 (0.06)**	−0.86 (0.08)***
Firstborn Characteristic	−0.0005 (0.15)	−0.13 (0.16)
**Model 4 Appetite (*n* = 90)**		
Intercept	1.95 (0.43)***	1.83 (0.46)***
Measurement time point (Secondborn age in years)	−0.21 (0.07)**	−0.90 (0.09)***
Firstborn Characteristic	−0.16 (0.07)*	−0.03 (0.07)
**Model 5 Rapid Weight Gain (*n* = 116)**		
Intercept	2.25 (0.20)***	2.32 (0.21)***
Measurement time point (Secondborn age in years)	−0.18 (0.06)**	−0.87 (0.08)***
Firstborn Characteristic	−0.09 (0.06)	0.07 (0.07)

† < 0.10,* < 0.05,** < 0.01,*** < 0.001

^a^Models adjusted for birth spacing, study group and FB characteristics

### Mothers’ perspectives on factors that influenced secondborn feeding: qualitative results

Qualitative findings helped to explain quantitative findings. In addition to firstborn child characteristics providing learning opportunities for mothers, *how* mothers learn from trusted sources and relationships is also important in their feeding practices. Themes detected from the interview data (example quotes provided in Table 6) provided further evidence that mothers’ experiences with firstborns had implications for their feeding practices with secondborns: Overall, mothers reported that they had gained knowledge and confidence about their feeding practices, which in turn affected their secondborn feeding practices. Three major themes were identified: 1) Use of feeding practices with secondborn that worked for the firstborn; 2) Confidence came from firstborn feeding experiences making secondborn feeding less anxiety-provoking; and 3) Additional experiences with firstborn and other factors that contributed to secondborn feeding practices.

**Table 6:** Additional quotes organized by themes pertaining to learned experience from semi-structured interviews with mothers

**Theme 1: Use of feeding practices with secondborn that worked for the firstborn**	
	A lot of what I learned from the older child [firstborn], I was able to apply to my younger child [secondborn] just having the confidence on you know, this is the schedule that works. Because what we found with our first child and then also with our second child is that routine is key so keep that schedule consistent. (ID 29)
	I think most of what we did with our younger [secondborn] was just based off of what we learned with our older [firstborn]. I think we did a lot of a similar things, you know, we started on the same type of foods and kind of food same way. (ID 22)
**Theme 2: Confidence came from firstborn feeding experiences making secondborn feeding less anxiety-provoking**	
	…it was just very I don’t even know. I guess we were just kind of wide eyed, didn’t really know what we were doing. You know, it was exciting, but it was also kind of terrifying because we were obviously new parents. We didn’t really know. (ID 13)
	I feel like once you learn it, you just are more confident. (ID 7)
**Theme 3: Additional experiences with firstborn and other factors that contributed to secondborn feeding practices**	
	I am a reader. I like research. If I had a question about something I would go get on the internet, which is maybe not the best thing, but I had books that were passed down or that my pediatrician would tell me to read. (ID 13)
	Having a baby, you only always want to have a baby, but you never really did any training for it, besides you read a lot of books, but the execution, you just have no idea what you’re getting yourself into. So to have someone [INSIGHT nurse] walk alongside me and say, can I show you something? For it to work, I was a believer from the minute I started it. (ID 2)
	Realistically with my first, and actually even my second, it was what worked for us. I did not do a lot of research online. I just went with my gut. (ID 20)
	It was a pretty easy transition. I guess like everything was still fresh in your mind and we knew how to do from the older one, so it’s just throwing an extra arm when you have a second one to juggle them. (ID 6)

#### Theme 1. Use of feeding practices with secondborn that worked for the firstborn

Mothers characterized their firstborn and secondborn children as different in terms of characteristics such as child temperament, appetite, and growth. Despite these sibling differences, the majority of mothers (22/30) reported using similar practices to feed their firstborns and secondborns. More specifically, mothers described two primary ways that they determined what feeding practices to use with their secondborn in infancy and toddlerhood. Mothers used similar feeding practices because they were perceived to be effective with firstborns and reflected on feeding practices learned at developmental milestones in firstborns (e.g., breastfeeding, introduction to solids) that they also applied to feeding secondborns. Mothers reported using similar feeding practices with firstborns and secondborns, regardless of differences in their characteristics, which is consistent with their learning from experience with their firstborns. For example, almost all mothers (29/30) reported using various structure-and control-based feeding practices with their firstborn children during toddlerhood – approaches that could then also used while feeding their secondborns.

“*We did use it* [food rewards] *for both... If he* [firstborn] *would try a new food that night, or if he would finish that new food that he was trying, we would do that. We also did reward them* [firstborn+secondborn] *for, you know, sitting quietly for five minutes so that I could have five minutes to myself... things like that..*.” (ID 18)

Mothers (20/30) also reported learning feeding practices at developmental milestones throughout infancy, such as breastfeeding or introduction to solid foods, while feeding their firstborn. This process of learning from their firstborn during these developmental milestones was described as facilitating feeding-related decisions when determining how to feed secondborns—additional evidence consistent with a learned experience process.

*“…it was helpful having a first child. I knew what to do and how to get her to latch on and I knew how to get her to you know, do the things I need to do, because I had experience with it with my first one.*” (ID 28)

The latter account about breastfeeding also highlights the importance of moving beyond measurement of firstborn characteristics to measure a broader range of maternal experiences with firstborns in efforts to understand the learned experience process.

#### Theme 2. Confidence came from firstborn feeding experiences making secondborn feeding less anxiety-provoking

Mothers described an overall smoother feeding experience with their secondborns in infancy and toddlerhood. They described less uncertainty about feeding their secondborn as an experienced mother as well as greater calm and confidence in feeding their secondborn because of their prior experiences. Mothers (16/30) discussed how their own characteristics such as their lack of experience and the emotions that come with being a new mother impacted their learned experience in feeding.

*“…when you're a first-time mom you think everything you're doing is wrong, even if it's not, you just do because you don't really know what you're doing.*” (ID 7)

As noted, mothers (17/30) also described feeling calmer and more confident about feeding their secondborns, especially as it related to their emotions and responses to their children.

“*I definitely remember feeling more relaxed. I'm not as stressed out about, you know, everything about having a second child. It didn’t all feel new so that was very helpful.*” (ID 12)

In sum, mothers’ explanations provide additional evidence of mothers’ learning from experiences with firstborns, expanding on the quantitative findings to illuminate factors beyond firstborn characteristics—particularly mothers’ experiences in firstborn feeding and psychosocial factors.

#### Theme 3. Additional experiences with firstborn and other factors that contributed to secondborn feeding practices

Mothers explained that they learned how to feed their secondborn children by gaining feeding-related knowledge based on firstborn experiences, recalling information and experiences that remained “fresh” from feeding their firstborn, receiving social support from their families and friends, and trusting their maternal instincts. First, most mothers (28/30) explained learning how to feed their firstborn in infancy and toddlerhood, with the help of parenting books and medical professionals.

“*The first one* [firstborn] *you're on the books, you're a bookworm…like you know everything. And then you're just trying to keep on doing what the book says, your doctor says, and stuff like that.*” (ID 10)

Second, mothers (12/30) reported how the spacing of their children’s births affected their feeding practices by allowing them to stay “fresh.” In this subsample, the mean birth spacing was28.7 months. Shorter birth spacing between siblings facilitated mothers’ application of their learned experiences because they could more easily remember what they learned in feeding their firstborn.

“*I kind of liked having them both closer in age where everything was still fresh in my mind from my older one* [firstborn] *when I had my younger one* [secondborn]*.*” (ID 15)

Third, mothers (10/30) explained the importance of social support as a facilitator of new knowledge acquisition in early motherhood. These mothers reported high levels of social support, such as marital partners, extended family members, and nurses from the INSIGHT study.

“*I was very lucky to have two older sisters that had just had kids, like a couple of months before me and one a year before me so I pretty much just relied on them for all information…*[with firstborn]*.*” (ID 13)

Finally, mothers (6/30) discussed their maternal instincts as drivers of feeding-related decisions.

“*Back then* [with firstborn]… *I could hear from the room away and know if it was a cry pain, if it was a cry of hunger, if it was cry of uncomfortable, if it was going to like end right away. It was just so instinctual, it was amazing… It comes so quickly.*” ( ID 19)

Together, responses relevant to this theme highlight four different ways that new mothers explained learning to feed their infants and toddlers.

## Discussion

The goal of this analysis was to elucidate whether and how learned experience processes are evident in maternal feeding practices in infancy and early childhood. A study strength was its use of longitudinal data to determine whether firstborn characteristics were associated with mothers’ feeding practices with secondborns over a three-year period of time. Although firstborn characteristics were not associated with mothers’ use of food to soothe with secondborns in infancy, firstborn appetite was a negative predictor of mothers’ food to soothe and firstborn negative affect was a positive predictor of pressure and a negative predictor of consistent mealtime routines when secondborns were toddlers. An additional contribution of this study was our use of semi-structured interviews to capture mothers’ perspectives on whether and how their experiences with their firstborns may have influenced their feeding practices with secondborns. Consistent with a learned experience process, mothers reported using feeding practices with their secondborn that they had learned with the firstborn, with respect to feeding milestones such as the transition to solid foods, and that their experiences with the firstborn had given them more confidence and knowledge about feeding practices. Beyond experiences with the firstborn, mothers also cited social support and maternal instinct as larger influences on their feeding practices. Below we elaborate on these findings, including their implications for future research and practice.

### Longitudinal links between firstborn characteristics and feeding practices with secondborns

Together, the findings provided support for the study hypotheses about the role of learned experience in mothers' feeding practices. As hypothesized (Hypothesis 1.i), quantitative findings indicated that firstborn negative affect was associated with some secondborn feeding practices. First, mothers of firstborns who were higher in negative affect used less consistent mealtime routines and more pressure to eat in feeding secondborns. Mothers whose infants are fussier may have more trouble establishing mealtime routines and thus find it more challenging to create mealtime routines with secondborns. Further, in dealing with a fussy firstborn, mothers may have learned the short-term benefits of pressuring their children to eat. Future research is needed to explicitly document such processes, but if observed, information about these processes may have implications for parent educational programs. Notably, these findings are consistent with studies that included only one child per family and showed that child negative affect was linked to greater use of food to soothe [21] and less mealtime structure [61] in early childhood. Ours, however, is the first analysis to use a sibling design to examine whether the effects of one child’s negative affect spill over to affect the feeding practices used with another.

In contrast to Hypothesis 1.ii, firstborns’ larger appetite predicted mothers’ less frequent use of food to soothe when secondborns were toddlers. Mothers may not have learned or needed to use food to soothe with their firstborns because their well-fed firstborns did not fuss as often. It also is possible that mothers learned how to soothe a fussy infant in ways other than food to soothe and used these strategies with their secondborns, or even that they became less reactive to fussy behaviors based on their experiences with firstborns and were more inclined to let their secondborns “cry it out.” Mothers’ reports in the interviews about their increased confidence are consistent with the latter interpretation. Prior studies report that child appetite is positively associated with food to soothe and with more restrictive parent feeding practices [25, 62]. Additionally, Hypothesis 1.iii was not supported by our data: firstborn rapid weight gain was not associated with feeding practices of secondborns in infancy or toddlerhood, a finding that is in line with other research showing that parental concern about child weight, but not weight status, was associated with child feeding [63]. These studies focus on only one child within a family, however, and thus more work is needed that uses a sibling design to test the learning from experience process.

### Exploration of mothers’ perspectives on factors that influenced their feeding of secondborns

Qualitative themes extended the quantitative findings about what mothers learn from their experiences with firstborns. In keeping with the null quantitative findings of firstborn characteristics and secondborn food to soothe, during the interviews, mothers did not mention learning about using food to soothe with secondborns in infancy, perhaps due to their focus on breast and other bottle feeding practices during this developmental stage [64, 65], which may be more relevant to mothers’ learning during infancy. Instead, qualitative themes highlighted the role of learning from experience in breastfeeding and in introduction of solids in infancy. In line with these findings, mothers in a similar qualitative study described feeling external pressures to start feeding solid foods to their infant [66], underscoring the importance of social influences on infant feeding. In another qualitative study, some mothers discussed learning from their first experience in breastfeeding and that they were more successful the second time [67] – consistent with mothers’ reports in the current analysis.

Although firstborn negative affect was associated with secondborn feeding in quantitative models, mothers did not describe this process explicitly in their interviews—underscoring the utility of methodological triangulation [68]. It may be that mothers were unaware of the role of the firstborns’ characteristics, despite probing, and that if asked more directly they may have acknowledged this or provided alternative explanations for the patterns of association we observed in the quantitative data—all directions for future study. Importantly, although most mothers discussed the characteristics of both of their children and that they used similar practices with each, they did not explicitly connect their feeding practices with specific child characteristics, suggesting that other contextual and psychosocial factors may play a larger role. Consistent with other parenting literature on learned experience [69, 70] mothers did, however, describe a general learning process: that feeding was not as difficult the second time because of what they had learned from their experiences with their firstborn. For example, mothers described their initial lack of experience in feeding their firstborn and that they were able to recall practices used with their firstborn and thus felt more confident feeding their secondborn. These findings are consistent with those of other studies showing that first-time mothers experience anxiety, labile emotions and stress over infant care [71] and that self-efficacy and postpartum anxiety are negatively associated with parity [72, 73] –results consistent with a learning from experience process.

Beyond their experiences with firstborns, mothers reported additional sources of knowledge about feeding: social support and maternal instinct. Mothers’ comments about social influences were consistent with findings from a similar study, that mothers learned from friends and family, formal information sources, and normative beliefs about food and feeding [74]. Also consistent with reports by mothers in our sample, another study of white, middle income first-time mothers found that family and social support were associated with less maternal maladjustment, and in turn, higher parental self-efficacy [75] –findings consistent with prior work showing that social support is linked to mothers’ success in breastfeeding [76, 77]. Future research should be directed toward the role of social support in other feeding practices. These psychosocial factors also provide a potential direction for family-focused obesity prevention.

Strengths of our analysis include the longitudinal, mixed methods, iterative design utilizing verbatim transcription as well as methodological and analytic triangulation. In the face of its contributions, limitations of our study imply additional directions for future research. First, the study sample was a mostly white, educated sample of mothers. Studying more diverse populations such as those closely connected to extended family may qualify our conclusions about the significance of firstborns in mothers’ learning. An additional limitation was the reliance on retrospective data in the semi-structured interviews as memory demands and response biases may have colored mothers’ reports. Research with mothers who are in the midst of learning to feed their firstborns may advance understanding of the learned experience process in real time. Finally, our subsample for the qualitative portion was drawn from a study related to siblings, which may have caused mothers to be more aware of their children’s differences, however, mothers reported similar feeding practices and experiences with their firstborn and secondborn child.

## Conclusions

This analysis is the first to examine a learned experience process in the context of maternal feeding in early childhood by testing whether firstborn characteristics predict secondborn feeding over time. The mixed methods design allowed for our use of qualitative data to expand on what we learned from quantitative analyses of firstborns’ role in secondborn feeding practices. Together, the findings suggest that mothers may develop specific feeding practices with their secondborn based on their experiences with their firstborns, including practices learned in the context of feeding firstborns and to a lesser extent, firstborn characteristics. Future studies should measure learning processes directly such as measuring mothers’ knowledge, feelings of confidence, and feeding skills prior to firstborns’ and again, prior to secondborns’ births. A variety of family structures (e.g., sibship size, sibling birth spacing) and family dynamics (e.g., involvement with extended family) should also be explored. Investigating larger family systems processes (e.g., coparenting) in addition to sibling-related processes may advance understanding of parental learning and practice of responsive feeding toward reducing the risk of childhood obesity and promoting child health.

## Data Availability

The datasets analyze during the current study are available from the senior author on reasonable request.

## Abbreviation

BMI: Body Mass Index
